# Influence of dietary supplementation with *Lepidium meyenii* (Maca) on sperm quality in dogs

**DOI:** 10.3389/fvets.2024.1375146

**Published:** 2024-02-29

**Authors:** Debora Teresa Gattuso, Angela Polisca, Claudia Dina Interlandi, Maria Rizzo, Marco Tabbì, Elisabetta Giudice, Santo Cristarella, Claudia Rifici, Marco Quartuccio, Viola Zappone

**Affiliations:** ^1^MGVet CSB (Canine Semen Bank), Reggio Calabria, Italy; ^2^Department of Veterinary Medicine, University of Perugia, Perugia, Italy; ^3^Department of Veterinary Sciences, University of Messina, Messina, Italy

**Keywords:** *Lepidium meyenii*, Maca, reproductive performance, Canine semen, dietary supplementation, male dogs

## Abstract

Maca is a traditional Andean crop used as a nutraceutical for the fertility-enhancing properties that are linked with antioxidant activity. The aim of this study was to evaluate, for the first time, the potential beneficial effects of oral Maca (*Lepidium meyenii* or *Lepidium peruvianum*) supplementation in improving reproductive performance in male dogs. Forty-eight male dogs of different breeds were enrolled in the study, fed the same maintenance diet, and exposed to the same environmental conditions. The subjects were divided into four groups of 12 dogs each: Subfertile treatment group, Subfertile control group, Normofertile treatment group, and Normofertile control group. The dogs in the treatment groups received *Lepidium meyenii* in their diet in a capsule formulation [75 mg/kg Maca extract 10:1, thickening agent (hydroxypropyl methylcellulose), ground rice], while the control groups received placebo capsule (starch). For each subject included in the study, the spermiogram was analyzed at three time points of the sperm cycle: at day 0 (T_0_), day 31 (T_31_), and day 62 (T_62_). Dietary supplementation with Maca in subfertile subjects resulted in a significant increase in ejaculate volume and total sperm count compared to the control group. This increase was also observed in normofertile subjects in the treatment group. In addition, total and progressive motility as well as sperm morphology were significantly improved in the groups treated with Maca compared to the control groups. The results thus highlight, for the first time, the potential efficacy of supplementation with 75 mg/kg of Maca extract daily in improving semen quality in dogs.

## Introduction

Male infertility is a widespread problem in dog breeding and can have serious financial implications (1). Infertility can be defined as the inability to mate or failure to fertilize after mating several fertile females (2). Such failure may be due to a lack of ejaculation or incomplete ejaculation caused by inadequate coital locking due to anxiety and discomfort during mating or sperm collection, or poor sperm quality (3).

Poor sperm quality can be caused by both congenital and acquired factors. Congenital factors, which are present from birth, are less common than acquired factors and are referred to as disorders of sexual development (DSD) (4). Acquired disorders, which develop during the dog’s lifetime (5, 6), can be the result of various factors including hormonal disorders, infectious diseases, stress, hyperthermia, nutritional deficiencies, exposure to toxins and autoimmune disorders (1, 3, 5–7).

During spermatogenesis and steroidogenesis, sperm accumulate reactive oxygen species (ROS) (8). Their activity is one of the major threats to sperm quality both *in vivo* and *in vitro* (9). Sperm function is not impaired when ROS and antioxidant levels are balanced, as this ensures that no significant damage occurs. However, metabolic oxidative stress caused by excessive ROS production or low antioxidant status, or both, can lead to impaired sperm function. Therefore, it is crucial to ensure low levels of ROS for proper fertilization, particularly for capacitation, hyperactivation and the acrosome reaction (10). Tafuri et al. (9) observed in stallions that Maca has a high antioxidant capacity that protects sperm and keeps ROS levels low.

*Lepidium meyenii*, commonly known as Maca, is a plant of the Brassicaceae family that is widely distributed in Peru, North America and Europe (11). It has become very popular due to its pharmacological properties, including antimicrobial, antioxidant, and anti-inflammatory activities (12, 13). In traditional medicine, this plant is known for its stimulating properties on fertility and sexual function, which is why it is often called the “Andean Viagra” (14). Maca is rich in valuable nutrients, including proteins, carbohydrates, essential amino acids, lipids, free fatty acids and various secondary metabolites such as macamides, alkaloids, and glucosinolates (14). The macamides and glucosinolates found in the plant reduce free radicals and protect cells from oxidative stress (15).

The effects of Maca on sperm quality, spermatogenesis, sperm count and sperm motility in different species have been described in several studies (16–18). These effects have been observed both in healthy animals (14, 16, 17, 19) and in animals with induced subfertility (20, 21).

Although much research has been carried out on the oral supplementation of Maca in different species, including humans (22, 23), its effects on the canine species have not yet been studied.

The aim of this study was to evaluate, for the first time, the potential beneficial effects of oral Maca supplementation in improving reproductive performance in male dogs.

## Materials and methods

### Ethical approval

All treatments, housing and animal care were in compliance with EU Directive 2010/63/EU on the protection of animals used for scientific purposes. The Ethics Committee of the Department of Veterinary Medicine and Animal Productions at the University of Messina, Italy (prot. no. 09/2023 ter), approved the protocol and procedures. Informed consent was obtained from each dog owner before its inclusion in the study.

### Animals and study design

A total of 48 client-owned healthy male dogs, heterogeneous in breed and age, were included in the study, which took place between April and November 2023. The subjects lived at home with their owners, were aged between 2 and 6 years, with a mean age of 3.84 ± 1.19 years, and their weight ranged from 25 to 62 Kg, with a mean of 35.95 ± 10.94 Kg. Inclusion criteria for the animals were based on clinical history, physical examination, reproductive ultrasound of the prostate and testes, and semen parameters such as ejaculate volume, total sperm concentration, total and progressive motility and morphology. To minimize defects in semen stored in the epididymis, such as reduced motility and increased debris, a preliminary semen collection was performed 48 h prior to the examination. Subjects had to be free of clinically relevant systemic and reproductive disorders, fed a specific commercial diet free of additives such as L-arginine and/or vitamins and antioxidants in doses that could interfere with the study. The maintenance diet for adult dogs had chicken as the first ingredient and its formulation was characterized by digestibility and palatability due to the inclusion of fresh meat. This diet contained 26% crude protein, 2.2% crude fiber, 14% crude fat, 6.5% crude ash, 1.2% calcium, 1% phosphorus, 0.7% n-3 fatty acids and 5.2% n-6 fatty acids. Finally, the animals had to be housed in rooms with adequate natural light.

Subjects who had semen parameters incompatible with adequate reproductive capacity and who had experienced at least one reproductive failure in the 6 months prior to the study, either by natural mating or artificial insemination, were classified as subfertile (*n* = 24).

Subjects with semen parameters compatible with adequate reproductive capacity and a normal reproductive history in the 6 months prior to the study, with numerically representative litter size by both natural mating and artificial insemination, were classified as normofertile (*n* = 24).

Subjects in both subfertile (*n* = 24) and normofertile (*n* = 24) groups were further divided into a control group (*n* = 12) and a treatment group (*n* = 12). The subjects were then divided into a subfertile control group (SC group; *n* = 12), a subfertile treatment group (ST group; *n* = 12), a normofertile control group (NC group; *n* = 12) and a normofertile treatment group (NT group; *n* = 12).

Subjects in the treatment groups (ST and NT groups) received an oral Maca supplement in capsule form at a dose of 75 mg/kg [Maca extract 10:1, thickening agent (5 gr of hydroxypropyl methylcellulose), 1 mg of ground rice]. The galenic preparation of the maca extract dietary supplement for canine use was carried out using crude black maca extract powder (Erbavoglio^™^). This extract is obtained by crushing and pulverizing the root of the plant. Subjects in the control groups (SC and NC groups) received a placebo consisting of a starch-only capsule.

Three semen samples were collected from each subject to assess ejaculate volume, total sperm concentration, total and progressive motility and morphology at three time points of the sperm cycle, for a total of 144 samples throughout the study. Samples were collected immediately before the start of oral supplementation (T_0_), after 31 days (T_31_) and after 62 days (T_62_).

### Sampling procedures and semen analysis

Sperm collection was performed in a quiet and appropriate environment with a non-slip floor, by manual collection and in the presence of a teasing bitch, after removal of the extragonadal reserve to minimize defects of sperm stored in the epididymis, such as reduced motility and increased debris.

The ejaculate was fractionated by discarding the third fraction and immediately examining the first two fractions. Motility was assessed both by light microscopy, using a slide on a thermostat with a 100x objective on which 10 μL of ejaculate was deposited, and by CASA software. Sperm concentration was assessed freshly with an SDM1 photometer (MiniTube^™^), by placing 10 μL of ejaculate in the appropriate loggia of the instrument’s analysis microscope. The analysis was performed with the following parameters: frame acquired 30, frame rate 60 Hz, minimum cell contrast 75, minimum cell size 4 pixels, straightness threshold 75%, path velocity threshold 100 μm/s − 1 average path velocity (VAP) cut-off 9.0 μm/s − 1, medium VAP cut-off 20 μm/s − 1, head size non-motile 4 pixels, head intensity non-motile 80, static head size 0.44–4.98, static head intensity 0.49–1.68 and static elongation 17–96%. After staining with eosin/nigrosine, cell morphology was assessed by examining at least 200 spermatozoa per slide.

### Statistical analysis

Two-way repeated measures analysis of variance (ANOVA) was applied to determine significant effect of supplementation (ST, SC, NT and NC groups) and time (T_0_, T_31_ and T_62_) on studied parameters. *p*-value <0.05 was considered statistically significant. Bonferroni’s multiple comparison test was applied for *post-hoc* comparison. Data were analyzed using statistical software Prism v. 5.01 (Graphpad Software Ltd., United States, 2007).

## Results

### Quantitative parameters

**Figure 1:**
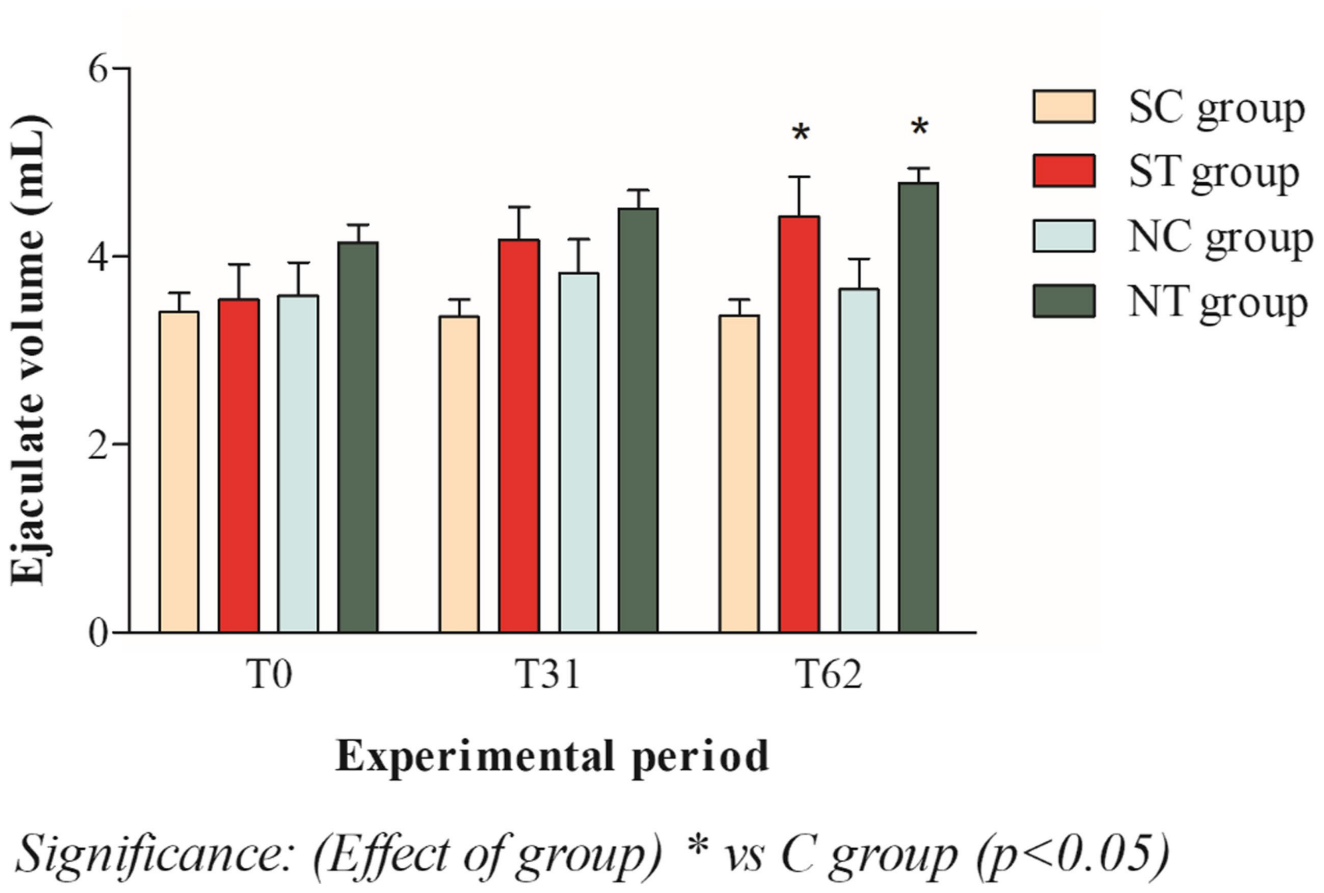
Ejaculate volume (mean ± SD) of semen collected at T_0_, T_31_, and T_62_ in subfertile treatment group (*n* = 12), subfertile control group (*n* = 12), normofertile treatment group (*n* = 12), and normofertile control group (*n* = 12; *p < 0.05).

The ejaculate volume at T_0_ of the subfertile subjects in the treatment group and in the control group was similar, with values of 3.57 ± 1.30 mL and 3.40 ± 0.69 mL, respectively. This parameter remained constant in the treatment group after oral Maca supplementation and showed a slight increase, reaching values of 4.17 ± 1.19 mL at T_31_ and 4.42 ± 1_._45 mL at T_62_ (*p* < 0.05). In the control group, on the other hand, this parameter did not change significantly, maintaining values of 3.35 ± 0.63 mL at T_31_ and 3.36 ± 0.59 mL at T_62_ (Figure 1).

**Figure 2:**
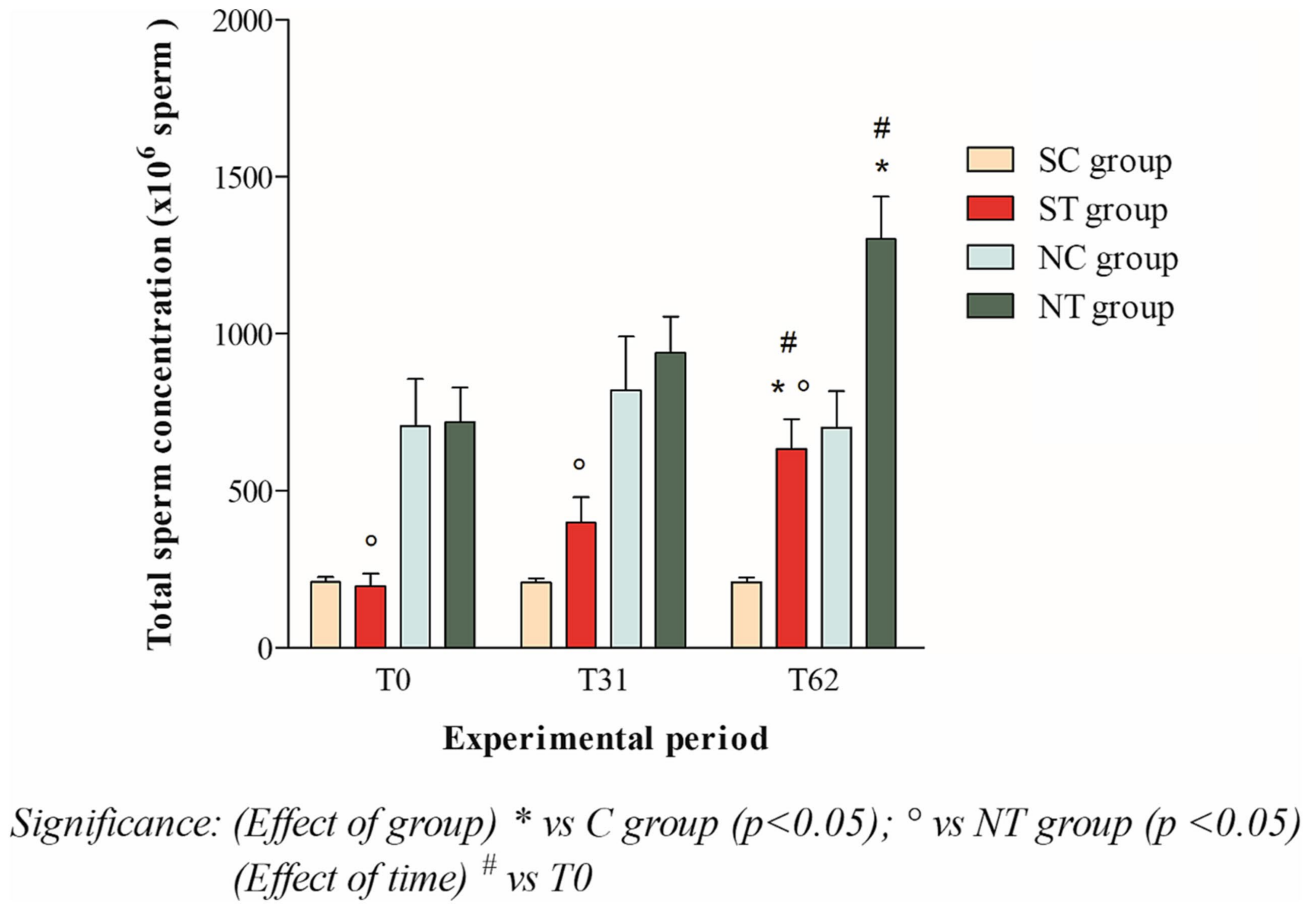
Total sperm count (mean ± SD) of semen collected at T_0_, T_31_, and T_62_ in subfertile treatment group (*n* = 12), subfertile control group (*n* = 12), normofertile treatment group (*n* = 12), and normofertile control group (*n* = 12; *p < 0.05).

At the beginning of the experimental design, the total sperm count of the two groups was also comparable. After oral Maca supplementation, the treatment group showed a significant increase from 196.87 ± 134.13 ×10^6^ spermatozoa (T_0_) to 632.21 ± 330.94 ×10^6^ spermatozoa (T_62_; *p* < 0.05). In the control group, the total sperm count remained essentially unchanged (208.43 ± 57.42 ×10^6^ spermatozoa at T_0_; 206.34 ± 52.34 ×10^6^ spermatozoa at T_31_; 207.66 ± 54.56 ×10^6^ spermatozoa at T_62_; Figure 2).

The ejaculate volume at T_0_ was also similar in subjects with normal fertility in the treatment and control groups, being 4.14 ± 0.65 mL and 3.58 ± 1.22 mL, respectively. This parameter remained constant in the treatment group after oral Maca supplementation and showed a slight increase, reaching values of 4.51 ± 0.67 mL at T_31_ and 4.77 ± 0.56 mL at T_62_ (*p* < 0.05). In the control group, on the other hand, this parameter did not change significantly, remaining at 3.82 ± 1.23 mL at T_31_ and 3.65 ± 1.13 mL at T_62_ (Figure 1).

In normofertile subjects in the treatment group, after oral Maca supplementation, the total sperm count showed a significant increase from 718.91 ± 377.48 ×10^6^ spermatozoa (T_0_) to 1301.52 ± 467.92 ×10^6^ spermatozoa (T_62_; *p* < 0.001). In the control group, the total sperm count remained essentially unchanged (706.01 ± 516.18 ×10^6^ spermatozoa at T_0_; 820.41 ± 589.15 ×10^6^ spermatozoa at T_31_ and 701.15 ± 400.88 ×10^6^ spermatozoa at T_62_; Figure 2).

Statistical comparison of ejaculate volume between the two treatment groups showed no significant difference at the three time points considered. However, the comparison of total sperm count showed a statistical difference at T_0_ (*p* < 0.01) and at T_31_ and T_62_ (*p* < 0.001).

### Motility and morphology

**Figure 3:**
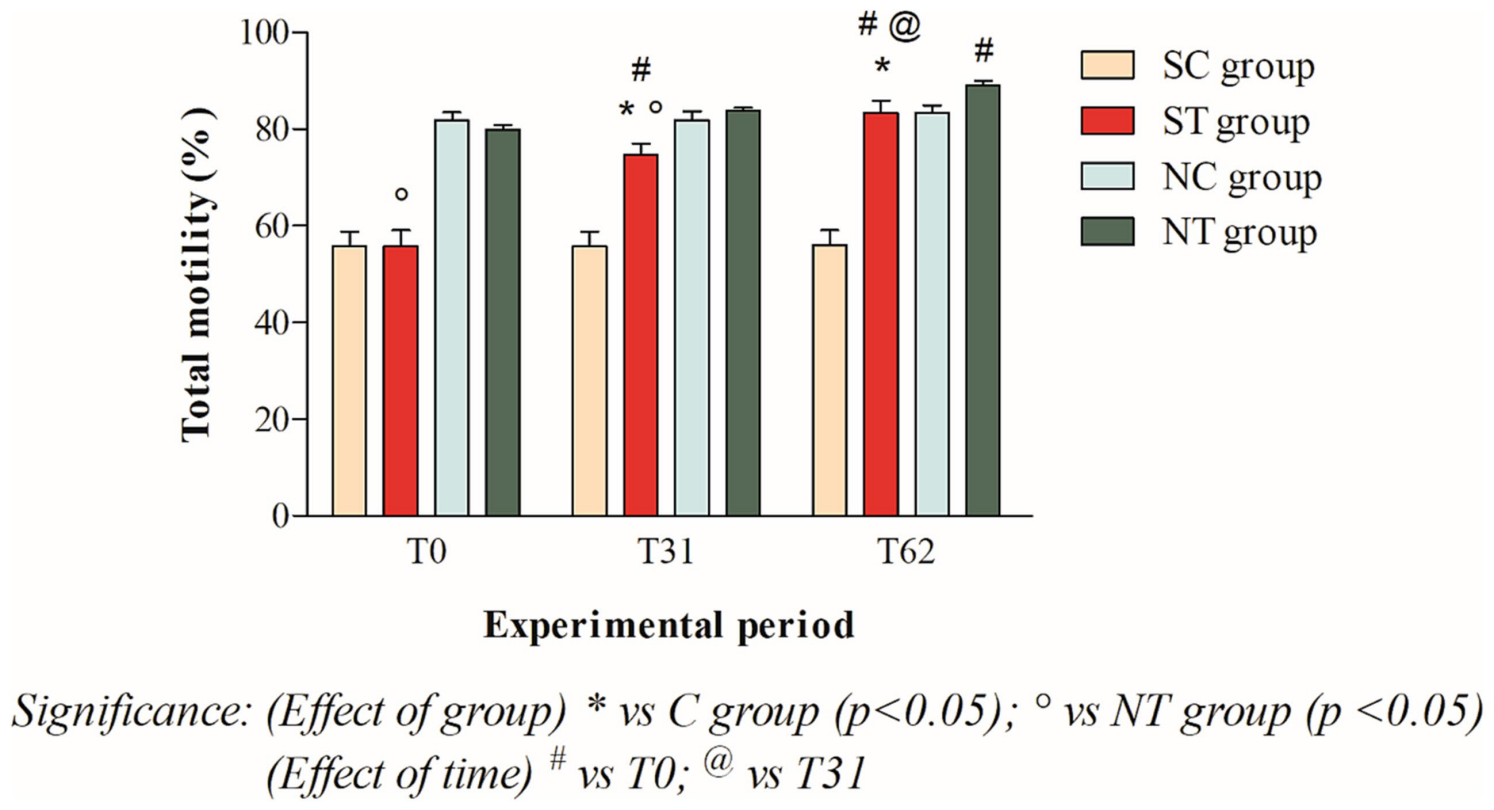
Total motility (mean ± SD) of semen collected at T_0_, T_31_, and T_62_ in subfertile treatment group (*n* = 12), subfertile control group (*n* = 12), normofertile treatment group (*n* = 12), and normofertile control group (*n* = 12; *p < 0.05).

The total motility at T_0_ of the subfertile subjects in the treatment and control groups was similar, at 55.75 ± 11.4% and 55.75 ± 10.65%, respectively. This parameter showed a significant increase in the treatment group after oral Maca supplementation, reaching values of 74.66 ± 7.8% at T_31_ (*p* < 0.001) and 83.41 ± 8.44% at T_62_ (*p* < 0.001). In the control group, however, this parameter did not change significantly, remaining at 55.66 ± 10.69% at T_31_ and 55.91 ± 10.92% at T_62_ (Figure 3).

**Figure 4:**
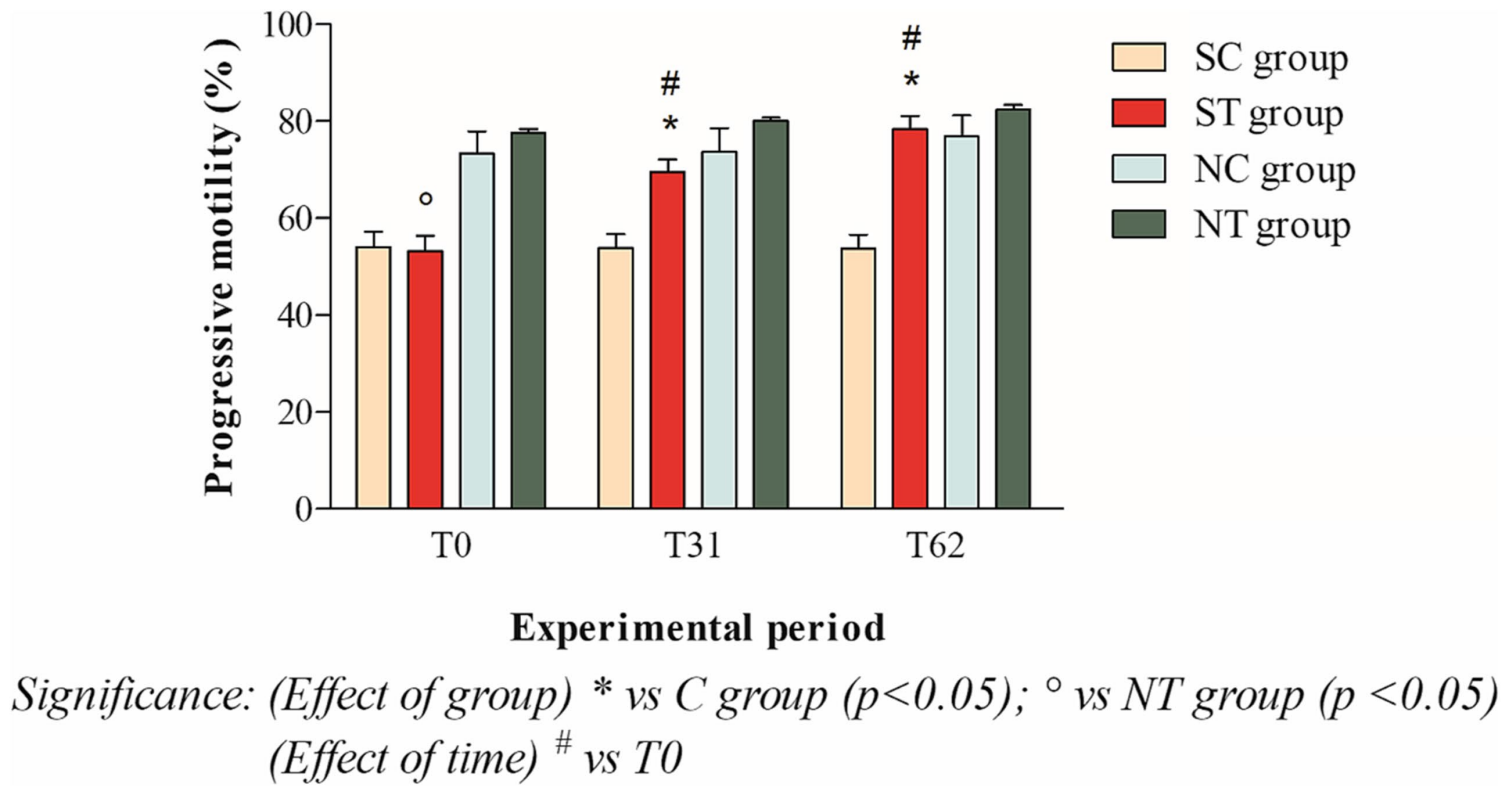
Progressive motility (mean ± SD) of semen collected at T_0_, T_31_, and T_62_ in subfertile treatment group (*n* = 12), subfertile control group (*n* = 12), normofertile treatment group (*n* = 12), and normofertile control group (*n* = 12; *p < 0.05).

The progressive motility at T_0_ of the subfertile subjects in the treatment and control groups was similar, with values of 53.16 ± 11.45% and 54.09 ± 10.71%, respectively. This parameter showed a significant increase in the treatment group after oral Maca supplementation, reaching values of 69.5 ± 8.98% at T_31_ (*p* < 0.001) and 78.33 ± 9.61% at T_62_ (*p* < 0.001). In the control group, however, this parameter did not change significantly, maintaining values of 53.80 ± 10.01% at T_31_ and 53.68 ± 10.29 at T_62_ (Figure 4).

**Figure 5:**
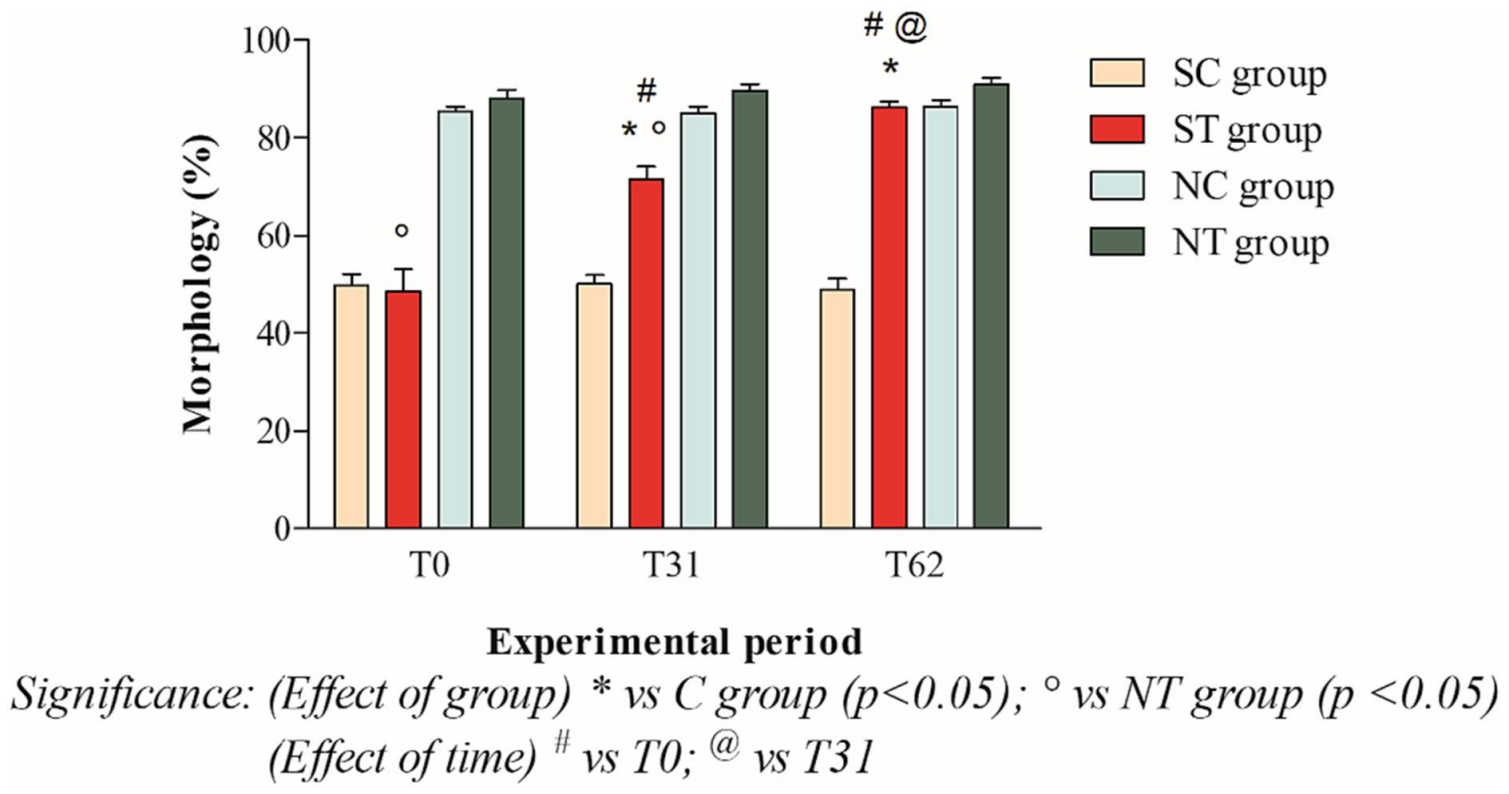
Morphology (mean ± SD) of semen collected at T_0_, T_31_, and T_62_ in subfertile treatment group (*n* = 12), subfertile control group (*n* = 12), normofertile treatment group (*n* = 12), and normofertile control group (*n* = 12; *p < 0.05).

At the beginning of the experimental design, the percentage of morphologically normal spermatozoa of the two groups was also comparable. After oral Maca supplementation, the treatment group showed a significant increase from 48.58 ± 15.87% (T_0_) to 86.08 ± 4.58 (T_62_; *p* < 0.001). In the control group, on the other hand, the percentages of morphology remained essentially unchanged (49.81 ± 8.05% at T_0_; 50.09 ± 6.24 at T_31_; 48.86 ± 8.19% at T_62_; Figure 5).

In subjects with normal fertility in the treatment and control groups, total motility was also similar at T_0_, with values of 79.91 ± 3.26% and 81.75 ± 6.0%, respectively. This parameter showed a slight increase in the treatment group after oral Maca supplementation, reaching values of 83.91 ± 1.88% at T_31_ and 89.75 ± 3.43% at T_62_. In the control group, on the other hand, this parameter did not change significantly, maintaining values of 73.66 ± 16.97% at T_31_ and 76.91 ± 14.93% at T_62_ (Figure 3).

In the normofertile subjects of the treatment and control groups, progressive motility was similar at T_0_, with values of 77.58 ± 2.77% and 73.33 ± 15.90%, respectively. This parameter showed a slight increase in the treatment group after oral Maca supplementation, reaching values of 80 ± 2.55% at T_31_ and 82.41 ± 3.31% at T_62_. In the control group, on the other hand, this parameter did not change significantly, maintaining values of 73.66 ± 16.97% at T_31_ and 76.91 ± 14.93% at T_62_ (Figure 4).

In normofertile subjects in the treatment group, after oral supplementation with Maca, the percentage of morphologically normal spermatozoa showed a slight increase from 88.16 ± 5.73% (T_0_) to 90.91 ± 4.66% (T_62_). In the control group, on the other hand, the morphology values remained essentially unchanged, with a slight increase (85.33 ± 3.74% at T_0_; 85 ± 4.89% at T_31_; 86.33 ± 4.83% at T_62_; Figure 5).

The comparison between the two treated groups showed a statistically significant difference in total motility at T_0_ (*p* < 0.001) and T_31_ (*p* < 0.05), but not at T_62_. In contrast, the comparison of progressive motility showed a statistically significant difference at T_0_ (*p* < 0.001) but not at T_31_ and T_62_. Finally, the comparison of morphology showed a statistically significant difference at T_0_ and T_31_ (*p* < 0.001), but not at T_62_.

## Discussion

The three main ecotypes of *Lepidium meyenii* are Yellow Maca, Red Maca and Black Maca, each of which has a different chemical composition and manifests different biological effects and medical applications. For example, Red Maca has been shown to reverse benign prostatic hyperplasia and osteoporosis experimentally induced in mice (24) and is effective in increasing sperm concentration (25). However, the most significant benefits on spermatogenesis were observed with the use of black and yellow maca. These two ecotypes were also effective in improving memory, with increased learning in mice (26). In addition, the use of black maca was associated with a reduction in glucose levels and a decrease in blood pressure (27).

Several studies, both *in vivo* and *in vitro*, have evaluated the antioxidant activity of Maca on different cell cultures, including macrophages, hepatocytes, and neurons. These studies have shown that Maca reduces free radicals, thus providing cytoprotection during oxidative stress (28–31). Although the mechanism of action of Maca is not fully understood, its protective role is probably due to increased superoxide dismutase enzyme activity (32). In a previous study, Vecera et al. (33) demonstrated a positive effect of Maca on systemic antioxidant status, with an improvement in ROS scavenger enzyme activity (superoxide dismutase, glutathione peroxidase and glutathione). An improvement in systemic antioxidant capacity after Maca supplementation explain the beneficial effect on fresh sperm quality.

Both yellow and black Maca improves male fertility by increasing sperm count and motility (27, 34) and promoting spermatogenesis (35, 36) without affecting hormone levels (17, 23). However, some *in vivo* studies in rats have reported an increase in testosterone levels after dietary supplementation with a powdered hydroalcoholic extract of Maca (37). The effect of Maca administration on the animal’s reproductive system and fertility has been associated with the plant lipid fraction, which contains macamides and primarily fatty acids (38, 39). Macamides may act directly on the reproductive tract by influencing the antioxidant balance (40).

The effects of using a maca plant powder or extract as a food supplement for humans have been studied in recent years, looking at the effects on reproductive traits in both men and women (22, 23). There is also growing interest in this product in veterinary medicine. Several studies have investigated its effects on ruminants (35, 36), showing that the administration of Maca extract as a dietary supplement induced an increase in semen quality and the number of copulations and ejaculations. Other similar studies have been carried out in stallions (17), rabbits (41), poultry (42, 43) and mice (16, 20, 21).

In mice and rats it has been found to increase male libido (39, 44), while in men it has shown promise in improving sexual desire without affecting hormone levels (45). Furthermore, a study in breeding bulls (46) showed positive results in both motility and sperm count after supplementation with Maca. Other research suggests that Maca may increase sperm production in men (11), increase testicular and epididymal weight in rats (16) and attenuate spermatogenetic disorders induced by high altitude conditions in rats (47). In addition, Maca supplementation has shown potential to improve spermatogenesis following damage from malathion poisoning (48).

The aim of this study was to evaluate, for the first time, the potential beneficial effects of oral Maca supplementation in improving reproductive performance in male dogs.

Ejaculate volume increased slightly and remained almost constant in the treatment groups during the experimental period. In contrast, total sperm count, total and progressive motility and morphology increased significantly at both T_31_ and T_62_, particularly in the subfertile group.

Total sperm production was approximately three times higher (*p* < 0.05) in subfertile subjects and two times higher (*p* < 0.001) in normo-fertile subjects at the end of the supplementation period than at the beginning. This result is in line with the literature, where this parameter was increased in studies conducted in stallions (17), adult rats (24, 25), peripubertal bulls (35) and men (11).

In both groups supplemented with Maca, increased total and progressive motility was observed, as well as increased morphology. This increase was not statistically significant in the normofertile group but was particularly marked in the subfertile group. The increases recorded allowed the subjects in the subfertile group to reach the physiological species ranges typical of the subjects in the normofertile group. This normalization is confirmed by the reduction in the statistical difference in the three parameters between the two treatment groups at different times. Total motility increased progressively, reaching the limit of statistical significance at T_31_ and normalization at T_62_. Morphology increased more slowly. It remained within the limits of statistical significance until T_31_ and normalized at T_62_. Progressive motility, on the other hand, increased sharply, reaching normalization at T_31_.

Although it was therefore necessary to reach T_62_ for all three parameters to reach physiological values overlapping with those recorded in subjects in the normofertile group, it is important to emphasize that progressive motility had already reached these values at T_31_.

The increase in sperm parameters in the normofertile subjects, although not statistically significant, and the normalization in the hypo-fertile subjects toward the physiological ranges of the species, confirm the effectiveness of oral Maca supplementation in improving reproductive performance.

Limitations of our study include the lack of assessment of hormonal levels in the experimental design to investigate the specific effects of Maca on these parameters, as well as the lack of specific tests to assess oxidative stress.

Further research is needed to verify and quantify the oral absorption of Maca in dogs and to clarify the mechanisms of *Lepidium meyenii*’s effects on sperm production. Future studies should evaluate the effects on additional sperm parameters such as membrane integrity and DNA fragmentation. They should also evaluate different times of treatment and different doses of supplementation.

## Conclusion

*Lepidium meyenii* has been used as a dietary supplement for its nutritional and therapeutic properties in several species, including humans. In the present study, we have shown for the first time that oral supplementation with 75 mg/kg of Maca extract in dogs can improve semen parameters such as ejaculate volume, total sperm count, total and progressive motility and morphology, resulting in improved reproductive performance.

## Data availability statement

The raw data supporting the conclusions of this article will be made available by the authors, without undue reservation.

## Ethics statement

The animal studies were approved by Ethics Committee of the Department of Veterinary Medicine and Animal Productions at the University of Messina, Italy (prot. no. 09/2023 ter). The studies were conducted in accordance with the local legislation and institutional requirements. Written informed consent was obtained from the owners for the participation of their animals in this study.

## Author contributions

DG: Conceptualization, Methodology, Writing – review & editing. AP: Methodology, Writing – review & editing. CI: Writing – original draft. MR: Formal analysis, Writing – review & editing. MT: Formal analysis, Writing – review & editing. EG: Conceptualization, Methodology, Writing – review & editing. SC: Writing – review & editing. CR: Writing – review & editing. MQ: Conceptualization, Writing – review & editing. VZ: Conceptualization, Formal analysis, Writing – original draft.
